# Patient reported outcome measures (PROMs) in cataract surgery: a systematic literature review protocol

**DOI:** 10.1186/s13643-026-03282-y

**Published:** 2026-08-18

**Authors:** Harry Rosen, Jan-Willem Verstraaten, Rustu Emre Akcan, Konrad Pesudovs, Frank Van den biggelaar, Mats Lundström, Lucia Brocato, Anders Behndig, Jos Kleijnen, Mor Dickman, ESCRS PROMs Task Force, Fabian Ekerhard, James Wolffsohn, Tanja Stamm, Katrin Wacker, ESCRS Registries Research Group, Adi Abulafia, Beatrice Cochener, Ype Henry, Madeleine Zetterberg, Marie Jose Tassingnon, Paul Rosen, Ulf Senevi

**Affiliations:** 1 Utrecht University, Utrecht, Netherlands; 2 Maastricht UMC+, Maastricht, Netherlands; 3 Koc University, Istanbul, Turkey; 4 University of New South Wales, Kensington, Australia; 5 Lund University, Lund, Sweden; 6 Royal College of Surgeons in Ireland, Dublin, Ireland; 7 Umeå University, Umeå, Sweden; 8 Kleijnen Systematic Reviews Ltd., Escrick, York, UK; 9 Utrecht UMC, Utrecht, Netherlands; 10 3 Royal Winchester Mews, Chilbolton Avenue, Winchester, SO22 5HX UK

**Keywords:** Patient reported outcome measures (PROMs), Patient reported outcomes (PROs), Cataract

## Abstract

**Background:**

Patient reported outcome measures (PROMs) are essential tools for capturing the quality of life and symptom burden from the patient’s perspective, particularly relevant in cataract surgery due to its subjective impact on vision. Cataract surgery, a highly cost-effective intervention, faces a growing demand for high-quality PROMs to measure diverse patient experiences and outcomes. Contemporary studies identify 14 domains of vision-related quality of life, such as convenience, visual symptoms, and driving, which traditional PROMs may inadequately address. Additionally, the changing cataract surgery landscape, driven by advancements in intraocular lenses (IOLs) and patient populations, necessitates a re-evaluation of PROMs for cataract patients.

**Methods:**

A systematic review will examine literature on PROMs in cataract surgery, including consensus studies, instrument development, psychometric evaluations, and implementation studies. Studies will be sourced from databases including PubMed, MEDLINE, Embase, Epub, CINAHL, CENTRAL, CDSR and Northern Light. Inclusion criteria focus on adult lens removal surgery, excluding non-ophthalmic, paediatric, or non-cataract studies. Articles will be screened by multiple reviewers. PROMs will be mapped to the 14 identified domains and evaluated using the COSMIN framework for psychometric assessment. Implementation aspects, including clinical usage, digitisation, and barriers to adoption, will also be assessed.

**Discussion:**

The review will address critical gaps in PROMs for cataract surgery, including domain coverage, psychometric quality, and implementation in clinical and academic settings. It will highlight the evolving needs of a diverse patient population, including refractive lens exchanges, and the impact of modern IOL technologies on PROMs. A well validated quality assessment of cataract PROMs will provide a strong platform to understand the established instruments. This comprehensive analysis will guide future research directions, including the development of new instruments or item banks, and inform best practices for PROM implementation, ensuring alignment with value-based healthcare principles.

**Systematic review registration:**

PROSPERO CRD420251007771

**Supplementary Information:**

The online version contains supplementary material available at https://doi.org/10.1186/s13643-026-03282-y.

## Background

Patient reported outcome measures (PROMs) are instruments which measure quality of life and symptom burden, directly from the perspective of the patients that experience them. They are invaluable tools for measuring the true benefit of medical interventions, particularly for a subjective sensation such as vision. Cataracts are a leading and growing cause of blindness worldwide [1–3], and surgical treatment has one of the most favourable cost-effectiveness profiles of any single medical intervention [4]. It is therefore paramount at both a global, and individualised perspective, that high quality PROMs exist for use in cataract surgery.

It is important to distinguish between patient reported outcomes (PROs) and PROMs. While PROs represent the construct being measured, PROMs are the actual instruments that are used to measure these constructs. There is no contemporary international consensus on the PROs that should be measured within a core outcome set for all cataract surgery. Groups such as the International Consortium for Health Outcomes Measurement (ICHOM), the Core Outcome Measures in Effectiveness Trials (COMET) and Tarricone et al. have attempted integrate PROMs [5–7]. Such initiatives have been limited by the availability of well validated PROMs for the domains relevant for modern cataract patients, and have utilised relatively little patient involvement. This literature review will therefore also identify what PROs have been identified, that are meaningful for patients.

Traditionally PROs and PROMs in cataract surgery have focussed on activity limitation as their primary QoL outcome measure [8–12]. Contemporary studies, however, have identified at least 14 different domains of vision related QoL [13–16], such as convenience, visual symptoms and driving etc. It is unclear whether PROMs in cataract surgery address these other domains.

Furthermore, it is unclear whether the available PROMs for cataract surgery are fit for purpose. PROMs must be psychometrically robust and specific enough for the chosen target population in which they are used [8–10, 17, 18]. In the case of patients undergoing cataract surgery, the population has become significantly more diverse since Lundstrom and Pesudovs published a review of cataract QoL questionnaires in 2011 [19]. In low- and middle-income countries (LMICs), or even some regions within high income countries, lens surgery is still a process of restoring vision and removing dense cataracts. However, in many high income countries, lens removal increasingly offers opportunities for refractive and presbyopic correction, representing a paradigm shift in cataract surgery [20, 21].

An emerging population of patients can undergo surgery with minimal lens opacity and good pre-operative visual acuity. This has been accelerated by developments in IOL technology offering improved intermediate capabilities or presbyopia correction. These IOL advances have placed concepts such as spectacle independence and dysphotopsias at the forefront of outcomes assessment research [22–28]. This paradigm shift in lens removal surgery demands a re-examination of current PROs and PROMs to ensure they are valid for the population in which they are used.

PROMs themselves have become increasingly advanced in recent years. The establishment of common metrics, item response theory (IRT), item banking and computer adaptive testing (CAT), are increasing our ability to create sensitive and psychometrically sound PROMs. Quality assessment of PROMs has also become more sophisticated, with well-established iterations of guidelines, having been developed by several different research groups [13, 29–31]. It is not clear how older PROMs for cataract surgery compare to modern quality assessment standards. Artificial intelligence has already been implemented in novel ways, and will inevitably play an increasing role in development, validation and interpretation of PROM scores, streamlining the cumbersome process of PROM validation [32].

Implementation factors are one of the primary barriers to the use of PROM questionnaires in routine clinical or academic practice. It is not clear how PROMs are being used at the point of care and what is being done with the outcome data. Factors such as practicality, convenience, digitisation, asynchrony or even literacy of the patients all impact uptake of PROMs and are essential to characterise to maximise the impact of PROMs. This is not only important clinically and academically but also at societal and regulatory levels for the purposes of making sound decisions based on the principles of value-based healthcare [33].

Timing between first and second eyes in cataract surgery is also a key factor that impacts patient reported QoL due to refractive imbalance. This is somewhat unique to ophthalmic surgery in QoL measurement due to our reliance on and habituation to binocular vision. This is important to identify in the literature and will form an important component of quality assessment in vision related QoL assessment.

In summary, there have been considerable recent changes in the cataract population, for whom the relevance of PROs has changed. Substantial advances have been made in IOL technology and the way we design and assess PROMs. There is therefore a strong demand for a contemporary, comprehensive review and evaluation of PROs and PROMs in cataract surgery. This will identify any gaps in the field and guide future directions for research, such as development of new instruments or item banks, and best practice for PROM implementation.

## Objectives

1. Identify a core outcome set of PROs meaningful for adult lens removal surgery from the perspective of patients and experts.
2. Identify PROM instruments currently used for adult lens removal surgery.
3. Evaluate the psychometric quality of PROM questionnaires for patients undergoing cataract surgery.
4. Compare PROs used in cataract surgery against previously identified 14 domains of ophthalmic related quality of life and map the PROMs to these domains
5. Summarise the current implementation of PROM questionnaires in cataract surgery.

PICO statement: this study aims to summarise and evaluate the PROs and PROMs used for patients with cataracts undergoing lens removal surgery.

## Methods

A systematic review of the literature will be conducted to identify PRO and PROM studies, including consensus, Delphi studies, instrument development, evaluation of measurement properties and implementation in real-world settings for lens removal surgery worldwide.

An international task force will be convened consisting of clinical experts, methods specialists, researchers from the public and private sectors and patient and industry representatives.

Studies will be excluded from the study if they are: animal studies, paediatric (< 18 years) populations, not about cataract (lens removal) surgery, combined cataract extraction with another procedure (e.g. glaucoma surgeries, retinal surgeries, intravitreal injections), physician surveys without representativeness, columns, lectures, correspondence, editorials or protocols. The study will endeavour to include all non-English studies, making use of AI translation software where necessary. Where unable to translate, studies will still be included up until the point of data extraction so that their existence can be acknowledged.

Search strategies were developed to identify relevant studies evaluating measurement properties, as recommended in the Centre for Reviews and Dissemination (CRD) guidance for undertaking reviews in health care [34] and the Cochrane Handbook for Systematic Reviews [35].

Candidate search terms were identified from target references, browsing database thesauri (e.g. MEDLINE MeSH and Embase EMTREE), existing reviews, and through consultation with the ESCRS guidelines team. Strategy development involved an iterative approach testing candidate text and indexing terms across a sample of bibliographic databases, aiming to reach a satisfactory balance of sensitivity and specificity. Search strategies were developed specifically for each database and the keywords and thesaurus terms were adapted according to the configuration of each database.

The search strategies comprised a facet for cataract surgery. This was then combined with generic terms for PROs, PROMs and health-related quality of life, as well as specific named outcome measures. Searches were not limited by date, language or publication status.

Databases included PubMed, MEDLINE(R) (Ovid) (including Epub Ahead of Print, In-Process, In-Data-Review and Other Non-Indexed Citations, Daily and Versions), Embase (Ovid), CINAHL (EBSCO), CENTRAL (Wiley), CDSR (Wiley) and Northern Light (Ovid). An example of our search strategy is included in the supplementary material. The search was carried out on the 5th November 2024, with a view to updating the search prior to publication, to identify any studies published in the elapsed interval period.

Database standardised search filters were used as denoted in the exclusion criteria where available. References identified from the searches were downloaded into Rayyan (https://www.rayyan.ai/) bibliographic management software for deduplication, further assessment and screening. Included articles, excluded reviews and author’s personal libraries were screened for relevant referenced articles meeting the inclusion criteria.

At least two authors will independently assess abstracts for inclusion and perform data extraction using predefined forms created in Microsoft Excel. Where disagreements are identified, full text articles will be reviewed. Further disagreement despite full text review will be resolved by mediation with a third reviewer.

Included studies will be initially categorised into three groups based on content:

1. Papers defining sets of PRO domains to be measured in cataract surgery
2. Papers assessing the measurement properties of PROMs in cataract surgery
3. Papers commenting on implementation or implementing PROMs themselves, i.e. using PROMs to measure a different outcome (e.g. in RCTs).

The second group will be further categorised by their target population, i.e. cataract surgery specific, ophthalmic (multiple eye disorders) or generic PROMs For the last group, only data regarding PROM implementation will be extracted. Where able, PROMs will be mapped against 14 previously identified ophthalmic QoL domains [13–16] and other sets of PRO domains. Quality of PROM items will be assessed based on the framework developed by the COSMIN group (COSMIN Risk of Bias Checklist, available from https://www.cosmin.nl/tools/guideline-conducting-systematic-review-outcome-measures/ accessed September 2024). Additional quality assessment items may be added in addition to this framework. These will be discussed and decided based on a consensus among the task force.

Examples of implementation data of interest to be identified will include clinical availability of PROMs and utilisation, use in industry, academic or audit utilisation, comparison with objective clinical measures, method of collection, e.g. digital or asynchronous, and convenience of the questionnaire. Implementation of PROMs will be discussed qualitatively as part of a separate body of work.

Prior to publication of this protocol, other major stakeholders such as industry partners and patient groups have been consulted. At least one patient representative will be involved in reading a lay summary of this protocol and providing feedback to ensure patient perspective remains at the heart of this work. Results will be published in a peer-reviewed scientific journal. Deviations from this protocol will be clearly documented and justified in the final publication. Meta-biases such as publication and reporting bias will be mitigated by various methods; including methodologist-led comprehensive multi-database and grey literature search, absence of language restrictions, adherence to internationally recognised gold standard frameworks and a-priori registration on PROSPERO.

## Supplementary Information

Supplementary Material 1: Appendix 1. Search strategies.

## Data Availability

The search strategy has been included as an appendix associated with this article for the purposes of reproducibility. Otherwise, no other data are applicable.

## Abbreviations

PROM: Patient reported outcome measure
PRO: Patient reported outcome
IOL: Intraocular lens
ICHOM: International Consortium for Health Outcomes Measurement
LMIC: Low and middle-income countries
EDOF: Extended depth of focus
IRT: Item response theory
CAT: Computer adaptive testing
CRD: Centre for Reviews and Dissemination
COSMIN: COnsensus-based Standards for the Selection of Health Measurement Instruments
