# Supplementary material for: Patient reported outcome measures (PROMs) in cataract surgery: a systematic literature review protocol
Source: Syst Rev. 2026 Aug 18;15:294. doi: 10.1186/s13643-026-03282-y (PMC13761103; doi:10.1186/s13643-026-03282-y)
Supplement: Supplementary file 1 [file 13643_2026_3282_MOESM1_ESM.docx]

**Resources searched:**

| **Resource** | **Host** | **Date range** | **Date searched** | **Records found** |
| --- | --- | --- | --- | --- |
| MEDLINE, Epub, DU & In-Process | Ovid | 1946 to 4.11.24 | 5.11.24 | 1355 |
| Embase | Ovid | 1974-4.11.24 | 5.11.24 | 1221 |
| Northern Light | Ovid | 2010-2024 Week 43 | 5.11.24 | 126 |
| CINAHL | EBSCO | 1981-5.11.24 | 5.11.24 | 190 |
| CENTRAL | Wiley | Issue 10 of 12, Oct 2024 | 5.11.24 | 351 |
| CDSR | Wiley | Issue 11 of 12, Nov 2024 | 5.11.24 | 105 |
| PubMed | https://pubmed.ncbi.nlm.nih.gov/ | to 5.11.24 | 5.11.24 | 475 |
| **TOTAL**  **(before deduplication)** |  |  |  | **3823** |
| **TOTAL**  **(after deduplication)** |  |  |  | **2298** |

Full details of all search strategies are presented in Appendix 1.

**APPENDIX 1 – SEARCH STRATEGIES**

**MEDLINE(R) and Epub Ahead of Print, In-Process, In-Data-Review & Other Non-Indexed Citations, Daily and Versions (Ovid): 1946 to November 04, 2024**

**Date searched: 5.11.24**

**Records found: 1355**

1	exp Cataract/su	191

2	exp Cataract Extraction/	38279

3	Ophthalmologic Surgical Procedures/ and cataract.ti,ab.	548

4	Lens Implantation, Intraocular/ and cataract.ti,ab.	7404

5	((cataract$ or lens or lenses or pseudoaphak$ or pseudo aphak$ or pseudophak$ or pseudo phak$) adj3 (extract$ or aspirat$ or operat$ or remov$ or surg$ or excis$ or implant$ or emulsif$ or cryoextract$)).ti,ab.	44042

6	((cataract$ or lens or lenses or pseudoaphak$ or pseudo aphak$ or pseudophak$ or pseudo phak$) adj3 (phakectom$ or zonulolys$ or capsulor?hexis or pha?oemulsif$ or pha?o emulsif$ or lensectom$ or capsulotom$)).ti,ab.	4397

7	((cataract$ or lens or lenses or pseudoaphak$ or pseudo aphak$ or pseudophak$ or pseudo phak$) adj3 (MSICS or ECCE or ICCE or SICS)).ti,ab.	787

8	(cataract$ adj3 (intraocular lens$ or implantable lens$ or IOL or IOLs)).ti,ab.	2694

9	cataractom$.ti,ab.	2

10	((lens$ or capsul$) adj4 (opaci$ or cloud$)).ti,ab.	6480

11	or/1-10	61169

12	Patient Reported Outcome Measures/	16612

13	((patient report$ or self report$) adj2 (outcome$ or assessment$ or symptom$ or experience$)).ti,ab.	61826

14	((patient report$ or self report$) adj2 (vision or visual or eyesight or sight or reading or health or wellness or activity or activities or satisfaction or dissatisfaction or happy or unhappy or happiness or unhappiness)).ti,ab.	18295

15	(PROM or PROMS or PREM or PREMS or HR-PRO or HRPRO).ti,ab.	9938

16	(Patient$ adj2 (self-report$ or self-assess$ or self-monitor$)).ti,ab.	9554

17	((Patient$ preference$ or patient$ satisfaction$) adj2 (assess$ or measure$ or tool$ or instrument$ or questionnaire$ or survey$ or score$ or scale$)).ti,ab.	12399

18	(Cat-PROM$ or Catquest$).ti,ab.	262

19	(National Eye Institute Visual Function Questionnaire or "NEI-VFQ-25").ti,ab.	902

20	patient health questionnaire/	1045

21	patient questionnaire$.ti,ab.	2791

22	quality-adjusted life years/ or quality of life/	309865

23	(sf36 or sf 36 or sf-36 or short form 36 or shortform 36 or sf thirtysix or sf thirty six or shortform thirtysix or shortform thirty six or short form thirty six or short form thirtysix or short form thirty six).ti,ab.	32426

24	(sf6 or sf 6 or sf-6 or short form 6 or shortform 6 or sf six or sfsix or shortform six or short form six).ti,ab.	2845

25	(sf12 or sf 12 or sf-12 or short form 12 or shortform 12 or sf twelve or sftwelve or shortform twelve or short form twelve).ti,ab.	8299

26	(sf6D or sf 6D or sf-6D or short form 6D or shortform 6D or sf six D or sfsixD or shortform six D or short form six D).ti,ab.	1063

27	(sf20 or sf 20 or sf-20 or short form 20 or shortform 20 or sf twenty or sftwenty or shortform twenty or short form twenty).ti,ab.	474

28	(sf8 or sf 8 or sf-8 or short form 8 or shortform 8 or sf eight or sfeight or shortform eight or short form eight).ti,ab.	799

29	("health related quality of life" or "vision related quality of life" or "vision specific quality of life").ti,ab.	65019

30	(Quality adjusted life or Quality-adjusted-life).ti,ab.	19064

31	"assessment of quality of life".ti,ab.	2374

32	(euroqol or euro qol or eq5d$ or eq 5d$).ti,ab.	19036

33	(hql or hrql or hqol or h qol or hrqol or hr qol).ti,ab.	30802

34	(hye or hyes).ti,ab.	77

35	health$ year$ equivalent$.ti,ab.	40

36	(hui or hui1 or hui2 or hui3 or hui4 or hui-4 or hui-1 or hui-2 or hui-3).ti,ab.	2135

37	(quality time or qwb or quality of well being or "quality of wellbeing" or "index of wellbeing" or "index of well being").ti,ab.	1300

38	(Disability adjusted life or Disability-adjusted life or health adjusted life or health-adjusted life or "years of healthy life" or healthy years equivalent or "years of potential life lost" or "years of health life lost").ti,ab.	7450

39	(QALY$ or DALY$ or HALY$ or YHL or HYES or YPLL or YHLL or qald$ or qale$ or qtime$ or AQoL$).ti,ab.	23196

40	(timetradeoff or time tradeoff or time trade-off or time trade off or TTO or Standard gamble$ or "willingness to pay").ti,ab.	12637

41	15d.ti,ab.	2059

42	(HSUV$ or health state$ value$ or health state$ preference$ or HSPV$).ti,ab.	544

43	(utilit$ adj3 ("quality of life" or valu$ or scor$ or measur$ or health or life or estimat$ or elicit$ or disease$)).ti,ab.	17844

44	(utilities or disutili$).ti,ab.	10650

45	or/12-44	471936

46	11 and 45	1390

47	(letter or editorial or note).pt.	1988167

**48	46 not 47	1355**

HRQoL free-text terms based on: Figure 4: Common free-text terms for electronic database searching for HSUVs in Papaioannou D, Brazier JE, Paisley S. NICE DSU Technical Support Document 9: the identification, review and synthesis of health state utility values from the literature (Internet), 2011 (accessed: 18.8.11) Available from: <http://www.nicedsu.org.uk>

**Embase (Ovid): 1974 to 2024 November 04**

**Date searched: 5.11.24**

**Records found: 1221**

1	exp *cataract/su	9510

2	exp *cataract extraction/	29793

3	*eye surgery/ and cataract.ti,ab.	925

4	exp *lens implant/ and cataract.ti,ab.	6966

5	((cataract$ or lens or lenses or pseudoaphak$ or pseudo aphak$ or pseudophak$ or pseudo phak$) adj3 (extract$ or aspirat$ or operat$ or remov$ or surg$ or excis$ or implant$ or emulsif$ or cryoextract$)).ti,ab.	52072

6	((cataract$ or lens or lenses or pseudoaphak$ or pseudo aphak$ or pseudophak$ or pseudo phak$) adj3 (phakectom$ or zonulolys$ or capsulor?hexis or pha?oemulsif$ or pha?o emulsif$ or lensectom$ or capsulotom$)).ti,ab.	5993

7	((cataract$ or lens or lenses or pseudoaphak$ or pseudo aphak$ or pseudophak$ or pseudo phak$) adj3 (MSICS or ECCE or ICCE or SICS)).ti,ab.	978

8	(cataract$ adj3 (intraocular lens$ or implantable lens$ or IOL or IOLs)).ti,ab.	3587

9	cataractom$.ti,ab.	1

10	((lens$ or capsul$) adj4 (opaci$ or cloud$)).ti,ab.	8011

11	or/1-10	65747

12	*patient-reported outcome/	20635

13	((patient report$ or self report$) adj2 (outcome$ or assessment$ or symptom$ or experience$)).ti,ab.	96261

14	((patient report$ or self report$) adj2 (vision or visual or eyesight or sight or reading or health or wellness or activity or activities or satisfaction or dissatisfaction or happy or unhappy or happiness or unhappiness)).ti,ab.	23958

15	(PROM or PROMS or PREM or PREMS or HR-PRO or HRPRO).ti,ab.	15790

16	(Patient$ adj2 (self-report$ or self-assess$ or self-monitor$)).ti,ab.	15650

17	((Patient$ preference$ or patient$ satisfaction$) adj2 (assess$ or measure$ or tool$ or instrument$ or questionnaire$ or survey$ or score$ or scale$)).ti,ab.	19014

18	(Cat-PROM$ or Catquest$).ti,ab.	298

19	(National Eye Institute Visual Function Questionnaire or "NEI-VFQ-25" or Patient-Reported Spectacle Independence Questionnaire or PRSIQ).ti,ab.	1242

20	exp *patient health questionnaire/	1162

21	patient questionnaire$.ti,ab.	4854

22	*quality adjusted life year/ or *quality of life index/	2571

23	Short Form 12/ or Short Form 20/ or Short Form 36/ or Short Form 8/	54455

24	"International Classification of Functioning, Disability and Health"/ or "ferrans and powers quality of life index"/	3933

25	(sf36 or sf 36 or sf-36 or short form 36 or shortform 36 or sf thirtysix or sf thirty six or shortform thirtysix or shortform thirty six or short form thirty six or short form thirtysix or short form thirty six).ti,ab.	52632

26	(sf6 or sf 6 or sf-6 or short form 6 or shortform 6 or sf six or sfsix or shortform six or short form six).ti,ab.	3166

27	(sf12 or sf 12 or sf-12 or short form 12 or shortform 12 or sf twelve or sftwelve or shortform twelve or short form twelve).ti,ab.	13123

28	(sf6D or sf 6D or sf-6D or short form 6D or shortform 6D or sf six D or sfsixD or shortform six D or short form six D).ti,ab.	1915

29	(sf20 or sf 20 or sf-20 or short form 20 or shortform 20 or sf twenty or sftwenty or shortform twenty or short form twenty).ti,ab.	552

30	(sf8 or sf 8 or sf-8 or short form 8 or shortform 8 or sf eight or sfeight or shortform eight or short form eight).ti,ab.	1264

31	("health related quality of life" or "vision related quality of life" or "vision specific quality of life").ti,ab.	93784

32	Quality adjusted life.ti,ab.	28906

33	"assessment of quality of life".ti,ab.	3808

34	(euroqol or euro qol or eq5d$ or eq 5d$).ti,ab.	34128

35	(hql or hrql or hqol or h qol or hrqol or hr qol).ti,ab.	49993

36	(hye or hyes).ti,ab.	195

37	health$ year$ equivalent$.ti,ab.	41

38	(hui or hui1 or hui2 or hui3 or hui4 or hui-4 or hui-1 or hui-2 or hui-3).ti,ab.	3432

39	(quality time or qwb or "quality of well being" or "quality of wellbeing" or "index of wellbeing" or index of well being).ti,ab.	1663

40	(Disability adjusted life or Disability-adjusted life or health adjusted life or health-adjusted life or "years of healthy life" or healthy years equivalent or "years of potential life lost" or "years of health life lost").ti,ab.	8910

41	(QALY$ or DALY$ or HALY$ or YHL or HYES or YPLL or YHLL or qald$ or qale$ or qtime$ or AQoL$).ti,ab.	38306

42	(timetradeoff or time tradeoff or time trade-off or time trade off or TTO or Standard gamble$ or "willingness to pay").ti,ab.	18729

43	15d.ti,ab.	3075

44	(HSUV$ or health state$ value$ or health state$ preference$ or HSPV$).ti,ab.	837

45	(utilit$ adj3 ("quality of life" or valu$ or scor$ or measur$ or health or life or estimat$ or elicit$ or disease$)).ti,ab.	28312

46	(utilities or disutili$).ti,ab.	17044

47	or/12-46	402893

**48	11 and 47	1221**

HRQoL free-text terms based on: Figure 4: Common free-text terms for electronic database searching for HSUVs in Papaioannou D, Brazier JE, Paisley S. NICE DSU Technical Support Document 9: the identification, review and synthesis of health state utility values from the literature (Internet), 2011 (accessed: 18.8.11) Available from: <http://www.nicedsu.org.uk>

**Northern Light Life Sciences Conference Abstracts (Ovid): 2010 - 2024 Week 43**

**Date searched: 5.11.24**

**Records found: 126**

1	((cataract$ or lens or lenses or pseudoaphak$ or pseudo aphak$ or pseudophak$ or pseudo phak$) adj3 (extract$ or aspirat$ or operat$ or remov$ or surg$ or excis$ or implant$ or emulsif$ or cryoextract$)).ti,ab.	10427

2	((cataract$ or lens or lenses or pseudoaphak$ or pseudo aphak$ or pseudophak$ or pseudo phak$) adj3 (phakectom$ or zonulolys$ or capsulor?hexis or pha?oemulsif$ or pha?o emulsif$ or lensectom$ or capsulotom$)).ti,ab.	793

3	((cataract$ or lens or lenses or pseudoaphak$ or pseudo aphak$ or pseudophak$ or pseudo phak$) adj3 (MSICS or ECCE or ICCE or SICS)).ti,ab.	80

4	(cataract$ adj3 (intraocular lens$ or implantable lens$ or IOL or IOLs)).ti,ab.	566

5	cataractom$.ti,ab.	0

6	((lens$ or capsul$) adj4 (opaci$ or cloud$)).ti,ab.	714

7	or/1-6	11211

8	((patient report$ or self report$) adj2 (outcome$ or assessment$ or symptom$ or experience$)).ti,ab.	10834

9	((patient report$ or self report$) adj2 (vision or visual or eyesight or sight or reading or health or wellness or activity or activities or satisfaction or dissatisfaction or happy or unhappy or happiness or unhappiness)).ti,ab.	1714

10	(PROM or PROMS or PREM or PREMS or HR-PRO or HRPRO).ti,ab.	1938

11	(Patient$ adj2 (self-report$ or self-assess$ or self-monitor$)).ti,ab.	1120

12	((Patient$ preference$ or patient$ satisfaction$) adj2 (assess$ or measure$ or tool$ or instrument$ or questionnaire$ or survey$ or score$ or scale$)).ti,ab.	1037

13	(Cat-PROM$ or Catquest$).ti,ab.	26

14	(National Eye Institute Visual Function Questionnaire or "NEI-VFQ-25").ti,ab.	78

15	patient questionnaire$.ti,ab.	191

16	(sf36 or sf 36 or sf-36 or short form 36 or shortform 36 or sf thirtysix or sf thirty six or shortform thirtysix or shortform thirty six or short form thirty six or short form thirtysix or short form thirty six).ti,ab.	1763

17	(sf6 or sf 6 or sf-6 or short form 6 or shortform 6 or sf six or sfsix or shortform six or short form six).ti,ab.	193

18	(sf12 or sf 12 or sf-12 or short form 12 or shortform 12 or sf twelve or sftwelve or shortform twelve or short form twelve).ti,ab.	442

19	(sf6D or sf 6D or sf-6D or short form 6D or shortform 6D or sf six D or sfsixD or shortform six D or short form six D).ti,ab.	154

20	(sf20 or sf 20 or sf-20 or short form 20 or shortform 20 or sf twenty or sftwenty or shortform twenty or short form twenty).ti,ab.	3

21	(sf8 or sf 8 or sf-8 or short form 8 or shortform 8 or sf eight or sfeight or shortform eight or short form eight).ti,ab.	38

22	("health related quality of life" or "vision related quality of life" or "vision specific quality of life").ti,ab.	8867

23	(Quality adjusted life or Quality-adjusted-life).ti,ab.	478

24	"assessment of quality of life".ti,ab.	268

25	(euroqol or euro qol or eq5d$ or eq 5d$).ti,ab.	1851

26	(hql or hrql or hqol or h qol or hrqol or hr qol).ti,ab.	8859

27	(hye or hyes).ti,ab.	285

28	health$ year$ equivalent$.ti,ab.	0

29	(hui or hui1 or hui2 or hui3 or hui4 or hui-4 or hui-1 or hui-2 or hui-3).ti,ab.	1000

30	(quality time or qwb or quality of well being or "quality of wellbeing" or "index of wellbeing" or "index of well being").ti,ab.	81

31	(Disability adjusted life or Disability-adjusted life or health adjusted life or health-adjusted life or "years of healthy life" or healthy years equivalent or "years of potential life lost" or "years of health life lost").ti,ab.	194

32	(QALY$ or DALY$ or HALY$ or YHL or HYES or YPLL or YHLL or qald$ or qale$ or qtime$ or AQoL$).ti,ab.	3118

33	(timetradeoff or time tradeoff or time trade-off or time trade off or TTO or Standard gamble$ or "willingness to pay").ti,ab.	1371

34	15d.ti,ab.	197

35	(HSUV$ or health state$ value$ or health state$ preference$ or HSPV$).ti,ab.	97

36	(utilit$ adj3 ("quality of life" or valu$ or scor$ or measur$ or health or life or estimat$ or elicit$ or disease$)).ti,ab.	2658

37	(utilities or disutili$).ti,ab.	1111

38	or/8-37	38442

**39	7 and 38	126**

**CINAHL (EBSCO)**

**Date searched: 5.11.24**

**Records found: 190**

S1	TI ( (cataract* or lens or lenses or pseudoaphak* or "pseudo aphak*" or pseudophak* or "pseudo phak*") N3 (extract* or aspirat* or operat* or remov* or surg* or excis* or implant* or emulsif* or cryoextract*) ) OR AB ( (cataract* or lens or lenses or pseudoaphak* or "pseudo aphak*" or pseudophak* or "pseudo phak*") N3 (extract* or aspirat* or operat* or remov* or surg* or excis* or implant* or emulsif* or cryoextract*) )	(6,721)

S2	TI ( (cataract* or lens or lenses or pseudoaphak* or "pseudo aphak*" or pseudophak* or "pseudo phak*") N3 (phakectom* or zonulolys* or capsulor?hexis or pha?oemulsif* or pha?o emulsif* or lensectom* or capsulotom*) ) OR AB ( (cataract* or lens or lenses or pseudoaphak* or "pseudo aphak*" or pseudophak* or "pseudo phak*") N3 (phakectom* or zonulolys* or capsulor?hexis or pha?oemulsif* or pha?o emulsif* or lensectom* or capsulotom*) )	(615)

S3	TI ( (cataract* or lens or lenses or pseudoaphak* or "pseudo aphak*" or pseudophak* or "pseudo phak*") N3 (MSICS or ECCE or ICCE or SICS) ) OR AB ( (cataract* or lens or lenses or pseudoaphak* or "pseudo aphak*" or pseudophak* or "pseudo phak*") N3 (MSICS or ECCE or ICCE or SICS) )	(107)

S4	TI ( cataract* N3 ("intraocular lens*" or "implantable lens*" or IOL or IOLs) ) OR AB ( cataract* N3 ("intraocular lens*" or "implantable lens*" or IOL or IOLs) )	(391)

S5	TI cataractom* OR AB cataractom*	(0)

S6	TI ( (lens* or capsul*) N4 (opaci* or cloud*) ) OR AB ( (lens* or capsul*) N4 (opaci* or cloud*) )	(514)

S7S1 OR S2 OR S3 OR S4 OR S5 OR S6	(7,076)

S8	TI ( ("patient report*" or "self report*") N2 (outcome* or assessment* or symptom* or experience*) ) OR AB ( ("patient report*" or "self report*") N2 (outcome* or assessment* or symptom* or experience*) )	(31,073)

S9	TI ( ("patient report*" or "self report*") N2 (vision or visual or eyesight or sight or reading or health or wellness or activity or activities or satisfaction or dissatisfaction or happy or unhappy or happiness or unhappiness) ) OR AB ( ("patient report*" or "self report*") N2 (vision or visual or eyesight or sight or reading or health or wellness or activity or activities or satisfaction or dissatisfaction or happy or unhappy or happiness or unhappiness) )	(11,480)

S10	TI ( PROM or PROMS or PREM or PREMS or HR-PRO or HRPRO ) OR AB ( PROM or PROMS or PREM or PREMS or HR-PRO or HRPRO )	(3,515)

S11	TI ( Patient* N2 ("self-report*" or "self-assess*" or "self-monitor*") ) OR AB ( Patient* N2 ("self-report*" or "self-assess*" or "self-monitor*") )	(5,113)

S12	TI ( ("Patient* preference*" or "patient* satisfaction*") N2 (assess* or measure* or tool* or instrument* or questionnaire* or survey* or score* or scale*) ) OR AB ( ("Patient* preference*" or "patient* satisfaction*") N2 (assess* or measure* or tool* or instrument* or questionnaire* or survey* or score* or scale*) )	(6,026)

S13	TI ( "Cat-PROM*" or Catquest* ) OR AB ( "Cat-PROM*" or Catquest* )	(25)

S14	TI ( "National Eye Institute Visual Function Questionnaire" or "NEI-VFQ-25" ) OR AB ( "National Eye Institute Visual Function Questionnaire" or "NEI-VFQ-25" )	(113)

S15	TI "patient questionnaire*" OR AB "patient questionnaire*"	(988)

S16	TI ( sf36 or "sf 36" or "sf-36" or "short form 36" or "shortform 36" or "sf thirtysix" or "sf thirty six" or "shortform thirtysix" or "shortform thirty six" or "short form thirty six" or "short form thirtysix" or "short form thirty six" ) OR AB ( sf36 or "sf 36" or "sf-36" or "short form 36" or "shortform 36" or "sf thirtysix" or "sf thirty six" or "shortform thirtysix" or "shortform thirty six" or "short form thirty six" or "short form thirtysix" or "short form thirty six" )	(12,001)

S17	TI ( sf6 or "sf 6" or "sf-6" or "short form 6" or "shortform 6" or "sf six" or sfsix or "shortform six" or "short form six" ) OR AB ( sf6 or "sf 6" or "sf-6" or "short form 6" or "shortform 6" or "sf six" or sfsix or "shortform six" or "short form six" )	(161)

S18	TI ( sf12 or "sf 12" or "sf-12" or "short form 12" or "shortform 12" or "sf twelve" or sftwelve or "shortform twelve" or "short form twelve" ) OR AB ( sf12 or "sf 12" or "sf-12" or "short form 12" or "shortform 12" or "sf twelve" or sftwelve or "shortform twelve" or "short form twelve" )	(3,359)

S19	TI ( sf6D or "sf 6D" or "sf-6D" or "short form 6D" or "shortform 6D" or "sf six D" or sfsixD or "shortform six D" or "short form six D" ) OR AB ( sf6D or "sf 6D" or "sf-6D" or "short form 6D" or "shortform 6D" or "sf six D" or sfsixD or "shortform six D" or "short form six D" )	(572)

S20	TI ( sf20 or "sf 20" or "sf-20" or "short form 20" or "shortform 20" or "sf twenty" or sftwenty or "shortform twenty" or "short form twenty" ) OR AB ( sf20 or "sf 20" or "sf-20" or "short form 20" or "shortform 20" or "sf twenty" or sftwenty or "shortform twenty" or "short form twenty" )	(74)

S21	TI ( sf8 or "sf 8" or "sf-8" or "short form 8" or "shortform 8" or "sf eight" or sfeight or "shortform eight" or "short form eight" ) OR AB ( sf8 or "sf 8" or "sf-8" or "short form 8" or "shortform 8" or "sf eight" or sfeight or "shortform eight" or "short form eight" )	(254)

S22	TI "health related quality of life" OR AB "health related quality of life" OR TI "vision related quality of life" OR AB "vision related quality of life" or TI "vision specific quality of life" OR AB "vision specific quality of life"	(29,326)

S23	TI ( "Quality adjusted life" or "Quality-adjusted-life" ) OR AB ( "Quality adjusted life" or "Quality-adjusted-life" )	(6,774)

S24	TI ( assessment of quality of life and psychological repercussions in women with endometriosis according to pain intensity. ) OR AB "assessment of quality of life"	(558)

S25	TI ( euroqol or "euro qol" or eq5d* or "eq 5d*" ) OR AB ( euroqol or "euro qol" or eq5d* or "eq 5d*" )	(2,892)

S26	TI ( hql or hrql or hqol or "h qol" or hrqol or "hr qol" ) OR AB ( hql or hrql or hqol or "h qol" or hrqol or "hr qol" )	(12,281)

S27	TI ( hye or hyes ) OR AB ( hye or hyes )	(9)

S28	TI "health* year* equivalent*" OR AB "health* year* equivalent*"	(5)

S29	TI ( hui or hui1 or hui2 or hui3 or hui4 or "hui-4" or "hui-1" or "hui-2" or "hui-3" ) OR AB ( hui or hui1 or hui2 or hui3 or hui4 or "hui-4" or "hui-1" or "hui-2" or "hui-3" )	(698)

S30	TI ( "quality time" or qwb or "quality of well being" or "quality of wellbeing" or "index of wellbeing" or "index of well being" ) OR AB ( "quality time" or qwb or "quality of well being" or "quality of wellbeing" or "index of wellbeing" or "index of well being" )	(576)

S31	TI ( "Disability adjusted life" or "Disability-adjusted life" or "health adjusted life" or "health-adjusted life" or "years of healthy life" or "healthy years equivalent" or "years of potential life lost" or "years of health life lost" ) OR AB ( "Disability adjusted life" or "Disability-adjusted life" or "health adjusted life" or "health-adjusted life" or "years of healthy life" or "healthy years equivalent" or "years of potential life lost" or "years of health life lost" )	(2,074)

S32	TI ( QALY* or DALY* or HALY* or YHL or HYES or YPLL or YHLL or qald* or qale* or qtime* or AQoL* ) OR AB ( QALY* or DALY* or HALY* or YHL or HYES or YPLL or YHLL or qald* or qale* or qtime* or AQoL* )	(7,509)

S33	TI ( timetradeoff or "time tradeoff" or "time trade-off" or "time trade off" or TTO or "Standard gamble*" or "willingness to pay" ) OR AB ( timetradeoff or "time tradeoff" or "time trade-off" or "time trade off" or TTO or "Standard gamble*" or "willingness to pay" )	(4,035)

S34	TI 15d OR AB 15d	(239)

S35	TI ( HSUV* or "health state* value*" or "health state* preference*" or HSPV* ) OR AB ( HSUV* or "health state* value*" or "health state* preference*" or HSPV* )	(247)

S36	TI ( utilit* N3 ("quality of life" or valu* or scor* or measur* or health or life or estimat* or elicit* or disease*) ) OR AB ( utilit* N3 ("quality of life" or valu* or scor* or measur* or health or life or estimat* or elicit* or disease*) )	(7,916)

S37	TI ( utilities or disutili* ) OR AB ( utilities or disutili* )	(62,774)

S38S8 OR S9 OR S10 OR S11 OR S12 OR S13 OR S14 OR S15 OR S16 OR S17 OR S18 OR S19 OR S20 OR S21 OR S22 OR S23 OR S24 OR S25 OR S26 OR S27 OR S28 OR S29 OR S30 OR S31 OR S32 OR S33 OR S34 OR S35 OR S36 OR S37	(159,460)

**S39	S7 AND S38	(190)**

**The Cochrane Library**

[**https://www.cochranelibrary.com/**](https://www.cochranelibrary.com/)

**Cochrane Database of Systematic Reviews (CDSR): Issue 11 of 12, November 2024**

**Date searched: 5.11.24**

**Records found: 105**

**Cochrane Central Register of Controlled Trials (CENTRAL): Issue 10 of 12, October 2024**

**Date searched: 5.11.24**

**Records found: 351**

#1	MeSH descriptor: [Cataract] this term only	2027

#2	MeSH descriptor: [Cataract Extraction] this term only	1767

#3	MeSH descriptor: [Ophthalmologic Surgical Procedures] this term only	507

#4	MeSH descriptor: [Lens Implantation, Intraocular] this term only	1575

#5	#3 or #4	2077

#6	cataract and #5	1435

#7	(cataract* or lens or lenses or pseudoaphak* or (pseudo NEXT aphak*) or pseudophak* or (pseudo NEXT phak*)) NEAR/3 (extract* or aspirat* or operat* or remov* or surg* or excis* or implant* or emulsif* or cryoextract*)	9131

#8	(cataract* or lens or lenses or pseudoaphak* or (pseudo NEXT aphak*) or pseudophak* or (pseudo NEXT phak*)) NEAR/3 (phakectom* or zonulolys* or capsulor?hexis or pha?oemulsif* or pha?o emulsif* or lensectom* or capsulotom*)	2194

#9	(cataract* or lens or lenses or pseudoaphak* or (pseudo NEXT aphak*) or pseudophak* or (pseudo NEXT phak*)) NEAR/3 (MSICS or ECCE or ICCE or SICS)	196

#10	cataract* NEAR/3 ((intraocular NEXT lens*) or (implantable NEXT lens*) or IOL or IOLs)	736

#11	cataractom*	0

#12	(lens* or capsul*) NEAR/4 (opaci* or cloud*)	936

#13	#1 or #2 or #6 or #7 or #8 or #9 or #10 or #11 or #12	9761

#14	MeSH descriptor: [Patient Reported Outcome Measures] this term only	1939

#15	((patient NEXT report*) or (self NEXT report*)) NEAR/2 (outcome* or assessment* or symptom* or experience*)	26669

#16	((patient NEXT report*) or (self NEXT report*)) NEAR/2 (vision or visual or eyesight or sight or reading or health or wellness or activity or activities or satisfaction or dissatisfaction or happy or unhappy or happiness or unhappiness)	5380

#17	PROM or PROMS or PREM or PREMS or HR-PRO or HRPRO	2514

#18	Patient* NEAR/2 ((self NEXT report*) or (self NEXT assess*) or (self NEXT monitor*))	3433

#19	((Patient* NEXT preference*) or (patient* NEXT satisfaction*)) NEAR/2 (assess* or measure* or tool* or instrument* or questionnaire* or survey* or score* or scale*)	11141

#20	(Cat NEXT PROM*) or Catquest*	40

#21	"National Eye Institute Visual Function Questionnaire" or "NEI-VFQ-25"	450

#22	MeSH descriptor: [Patient Health Questionnaire] this term only	122

#23	(patient NEXT questionnaire*)	1358

#24	MeSH descriptor: [Quality-Adjusted Life Years] this term only	2432

#25	MeSH descriptor: [Quality of Life] this term only	45177

#26	sf36 or "sf 36" or "sf-36" or "short form 36" or "shortform 36" or "sf thirtysix" or "sf thirty six" or "shortform thirtysix" or "shortform thirty six" or "short form thirty six" or "short form thirtysix" or "short form thirty six"	21007

#27	sf6 or "sf 6" or "sf-6" or "short form 6" or "shortform 6" or "sf six" or sfsix or "shortform six" or "short form six"	366

#28	sf12 or "sf 12" or "sf-12" or "short form 12" or "shortform 12" or "sf twelve" or sftwelve or "shortform twelve" or "short form twelve"	4709

#29	sf6D or "sf 6D" or "sf-6D" or "short form 6D" or "shortform 6D" or "sf six D" or sfsixD or "shortform six D" or "short form six D"	404

#30	sf20 or "sf 20" or "sf-20" or "short form 20" or "shortform 20" or "sf twenty" or sftwenty or "shortform twenty" or "short form twenty"	143

#31	sf8 or "sf 8" or "sf-8" or "short form 8" or "shortform 8" or "sf eight" or sfeight or "shortform eight" or "short form eight"	397

#32	"health related quality of life" or "vision related quality of life" or "vision specific quality of life"	27171

#33	"Quality adjusted life" or "Quality-adjusted-life"	7592

#34	"assessment of quality of life"	1085

#35	euroqol or "euro qol" or eq5d* or (eq NEXT 5d*)	16559

#36	hql or hrql or hqol or "h qol" or hrqol or "hr qol"	10426

#37	hye or hyes	131

#38	(health* NEXT year* NEXT equivalent*)	2

#39	hui or hui1 or hui2 or hui3 or hui4 or "hui-4" or "hui-1" or "hui-2" or "hui-3"	2605

#40	"quality time" or qwb or "quality of well being" or "quality of wellbeing" or "index of wellbeing" or "index of well being"	320

#41	"Disability adjusted life" or "Disability-adjusted life" or "health adjusted life" or "health-adjusted life" or "years of healthy life" or "healthy years equivalent" or "years of potential life lost" or "years of health life lost"	639

#42	QALY* or DALY* or HALY* or YHL or HYES or YPLL or YHLL or qald* or qale* or qtime* or AQoL*	8002

#43	timetradeoff or "time tradeoff" or "time trade-off" or "time trade off" or TTO or (Standard NEXT gamble*) or "willingness to pay"	2744

#44	15d	372

#45	HSUV* or (health NEXT state* NEXT value*) or (health NEXT state* NEXT preference*) or HSPV*	280

#46	utilit* NEAR/3 ("quality of life" or valu* or scor* or measur* or health or life or estimat* or elicit* or disease*)	4157

#47	utilities or disutili*	1707

#48	#14 or #15 or #16 or #17 or #18 or #19 or #20 or #21 or #22 or #23 or #24 or #25 or #26 or #26 or #28 or #29 or #30 or #31 or #32 or #33 or #34 or #35 or #36 or #37 or #38 or #39 or #40 or #41 or #42 or #43 or #44 or #45 or #46 or #47	136609

#49	#13 and #48	564

**#50	#49 in Cochrane Reviews	105 [CDSR]**

#51	#49 in Trials	444

#52	(trial registry record or Clinical trial protocol):pt	540744

**#53	#51 not #52	351 [CENTRAL]**

**PubMed:** [**https://pubmed.ncbi.nlm.nih.gov/**](https://pubmed.ncbi.nlm.nih.gov/)

**Date searched: 5.11.24**

**Records found: 475**

- This search was designed to identify only those records which would not have been retrieved by the Ovid MEDLINE searches, due to either a delay in publication on Ovid or because of the discrepancy between MEDLINE content on PubMed and Ovid.

| 1 | ((cataract*[Title/Abstract] OR lens[Title/Abstract] OR lenses[Title/Abstract] OR pseudoaphak*[Title/Abstract] OR pseudo aphak*[Title/Abstract] OR pseudophak*[Title/Abstract] OR pseudo phak*[Title/Abstract]) AND (extract*[Title/Abstract] OR aspirat*[Title/Abstract] OR operat*[Title/Abstract] OR remov*[Title/Abstract] OR surg*[Title/Abstract] OR excis*[Title/Abstract] OR implant*[Title/Abstract] OR emulsif*[Title/Abstract] OR cryoextract*[Title/Abstract])) | 68,036 |
| --- | --- | --- |
| 2 | ((cataract*[Title/Abstract] OR lens[Title/Abstract] OR lenses[Title/Abstract] OR pseudoaphak*[Title/Abstract] OR pseudo aphak*[Title/Abstract] OR pseudophak*[Title/Abstract] OR pseudo phak*[Title/Abstract]) AND (phakectom*[Title/Abstract] OR zonulolys*[Title/Abstract] OR capsulor*hexis[Title/Abstract] OR pha*oemulsif*[Title/Abstract] OR pha*o emulsif*[Title/Abstract] OR lensectom*[Title/Abstract] OR capsulotom*[Title/Abstract])) | 4,522 |
| 3 | ((cataract*[Title/Abstract] OR lens[Title/Abstract] OR lenses[Title/Abstract] OR pseudoaphak*[Title/Abstract] OR pseudo aphak*[Title/Abstract] OR pseudophak*[Title/Abstract] OR pseudo phak*[Title/Abstract]) AND (MSICS[Title/Abstract] OR ECCE[Title/Abstract] OR ICCE[Title/Abstract] OR SICS[Title/Abstract])) | 981 |
| 4 | (cataract*[Title/Abstract] AND (intraocular lens*[Title/Abstract] OR implantable lens*[Title/Abstract] OR IOL[Title/Abstract] OR IOLs[Title/Abstract])) | 12,541 |
| 5 | cataractom*[Title/Abstract] | 2 |
| 6 | ((lens*[Title/Abstract] OR capsul*[Title/Abstract]) AND (opaci*[Title/Abstract] OR cloud*[Title/Abstract])) | 8,818 |
| 7 | #1 or #2 or #3 or #4 or #5 or #6 | 72,835 |
| 8 | ((patient report*[Title/Abstract] OR self report*[Title/Abstract]) AND (outcome*[Title/Abstract] OR assessment*[Title/Abstract] OR symptom*[Title/Abstract] OR experience*[Title/Abstract])) | 192,725 |
| 9 | ((patient report*[Title/Abstract] OR self report*[Title/Abstract]) AND (vision[Title/Abstract] OR visual[Title/Abstract] OR eyesight[Title/Abstract] OR sight[Title/Abstract] OR reading[Title/Abstract] OR health[Title/Abstract] OR wellness[Title/Abstract] OR activity[Title/Abstract] OR activities[Title/Abstract] OR satisfaction[Title/Abstract] OR dissatisfaction[Title/Abstract] OR happy[Title/Abstract] OR unhappy[Title/Abstract] OR happiness[Title/Abstract] OR unhappiness[Title/Abstract])) | 158,126 |
| 10 | (PROM[Title/Abstract] OR PROMS[Title/Abstract] OR PREM[Title/Abstract] OR PREMS[Title/Abstract] OR HR-PRO[Title/Abstract] OR HRPRO[Title/Abstract]) | 10,950 |
| 11 | (Patient*[Title/Abstract] AND (self-report*[Title/Abstract] OR self-assess*[Title/Abstract] OR self-monitor*[Title/Abstract])) | 85,097 |
| 12 | ((Patient* preference*[Title/Abstract] OR patient* satisfaction*[Title/Abstract]) AND (assess*[Title/Abstract] OR measure*[Title/Abstract] OR tool*[Title/Abstract] OR instrument*[Title/Abstract] OR questionnaire*[Title/Abstract] OR survey*[Title/Abstract] OR score*[Title/Abstract] OR scale*[Title/Abstract])) | 55,111 |
| 13 | (Cat-PROM*[Title/Abstract] OR Catquest*[Title/Abstract]) | 218 |
| 14 | (National Eye Institute Visual Function Questionnaire[Title/Abstract] OR "NEI-VFQ-25"[Title/Abstract]) | 907 |
| 15 | patient questionnaire*[Title/Abstract] | 2,810 |
| 16 | (sf36[Title/Abstract] OR sf 36[Title/Abstract] OR sf-36[Title/Abstract] OR short form 36[Title/Abstract] OR shortform 36[Title/Abstract] OR sf thirtysix[Title/Abstract] OR sf thirty six[Title/Abstract] OR shortform thirtysix[Title/Abstract] OR shortform thirty six[Title/Abstract] OR short form thirty six[Title/Abstract] OR short form thirtysix[Title/Abstract] OR short form thirty six[Title/Abstract]) | 32,892 |
| 17 | (sf6[Title/Abstract] OR sf 6[Title/Abstract] OR sf-6[Title/Abstract] OR short form 6[Title/Abstract] OR shortform 6[Title/Abstract] OR sf six[Title/Abstract] OR sfsix[Title/Abstract] OR shortform six[Title/Abstract] OR short form six[Title/Abstract]) | 7,450 |
| 18 | (sf12[Title/Abstract] OR sf 12[Title/Abstract] OR sf-12[Title/Abstract] OR short form 12[Title/Abstract] OR shortform 12[Title/Abstract] OR sf twelve[Title/Abstract] OR sftwelve[Title/Abstract] OR shortform twelve[Title/Abstract] OR short form twelve[Title/Abstract]) | 9,612 |
| 19 | (sf6D[Title/Abstract] OR sf 6D[Title/Abstract] OR sf-6D[Title/Abstract] OR short form 6D[Title/Abstract] OR shortform 6D[Title/Abstract] OR sf six D[Title/Abstract] OR sfsixD[Title/Abstract] OR shortform six D[Title/Abstract] OR short form six D[Title/Abstract]) | 1,082 |
| 20 | (sf20[Title/Abstract] OR sf 20[Title/Abstract] OR sf-20[Title/Abstract] OR short form 20[Title/Abstract] OR shortform 20[Title/Abstract] OR sf twenty[Title/Abstract] OR sftwenty[Title/Abstract] OR shortform twenty[Title/Abstract] OR short form twenty[Title/Abstract]) | 6,066 |
| 21 | (sf8[Title/Abstract] OR sf 8[Title/Abstract] OR sf-8[Title/Abstract] OR short form 8[Title/Abstract] OR shortform 8[Title/Abstract] OR sf eight[Title/Abstract] OR sfeight[Title/Abstract] OR shortform eight[Title/Abstract] OR short form eight[Title/Abstract]) | 8,420 |
| 22 | ("health related quality of life"[Title/Abstract] OR "vision related quality of life"[Title/Abstract] OR "vision specific quality of life"[Title/Abstract]) | 66,978 |
| 23 | (Quality adjusted life[Title/Abstract] OR Quality-adjusted-life[Title/Abstract]) | 19,304 |
| 24 | "assessment of quality of life"[Title/Abstract] | 1,993 |
| 25 | (euroqol[Title/Abstract] OR euro qol[Title/Abstract] OR eq5d*[Title/Abstract] OR eq 5d*[Title/Abstract]) | 19,151 |
| 26 | (hql[Title/Abstract] OR hrql[Title/Abstract] OR hqol[Title/Abstract] OR h qol[Title/Abstract] OR hrqol[Title/Abstract] OR hr qol[Title/Abstract]) | 31,266 |
| 27 | (hye[Title/Abstract] OR hyes[Title/Abstract]) | 83 |
| 28 | health* year* equivalent*[Title/Abstract] | 40 |
| 29 | (hui[Title/Abstract] OR hui1[Title/Abstract] OR hui2[Title/Abstract] OR hui3[Title/Abstract] OR hui4[Title/Abstract] OR hui-4[Title/Abstract] OR hui-1[Title/Abstract] OR hui-2[Title/Abstract] OR hui-3[Title/Abstract]) | 2,172 |
| 30 | (quality time[Title/Abstract] OR qwb[Title/Abstract] OR quality of well being[Title/Abstract] OR "quality of wellbeing"[Title/Abstract] OR "index of wellbeing"[Title/Abstract] OR "index of well being"[Title/Abstract]) | 912 |
| 31 | (Disability adjusted life[Title/Abstract] OR Disability-adjusted life[Title/Abstract] OR health adjusted life[Title/Abstract] OR health-adjusted life[Title/Abstract] OR "years of healthy life"[Title/Abstract] OR healthy years equivalent[Title/Abstract] OR "years of potential life lost"[Title/Abstract] OR "years of health life lost"[Title/Abstract]) | 7,535 |
| 32 | (QALY*[Title/Abstract] OR DALY*[Title/Abstract] OR HALY*[Title/Abstract] OR YHL[Title/Abstract] OR HYES[Title/Abstract] OR YPLL[Title/Abstract] OR YHLL[Title/Abstract] OR qald*[Title/Abstract] OR qale*[Title/Abstract] OR qtime*[Title/Abstract] OR AQoL*[Title/Abstract]) | 23,548 |
| 33 | (timetradeoff[Title/Abstract] OR time tradeoff[Title/Abstract] OR time trade-off[Title/Abstract] OR time trade off[Title/Abstract] OR TTO[Title/Abstract] OR Standard gamble*[Title/Abstract] OR "willingness to pay"[Title/Abstract]) | 12,845 |
| 34 | 15d[Title/Abstract] | 2,370 |
| 35 | (HSUV*[Title/Abstract] OR health state* value*[Title/Abstract] OR health state* preference*[Title/Abstract] OR HSPV*[Title/Abstract]) | 543 |
| 36 | (utilit*[Title/Abstract] AND ("quality of life"[Title/Abstract] OR valu*[Title/Abstract] OR scor*[Title/Abstract] OR measur*[Title/Abstract] OR health[Title/Abstract] OR life[Title/Abstract] OR estimat*[Title/Abstract] OR elicit*[Title/Abstract] OR disease*[Title/Abstract])) | 186,492 |
| 37 | (utilities[Title/Abstract] OR disutili*[Title/Abstract]) | 10,758 |
| 38 | #8 or #9 or #10 or #11 or #12 or #13 or #14 or #15 or #16 or #17 or #18 or #19 or #20 or #21 or #22 or #23 or #24 or #25 or #26 or #27 or #28 or #29 or #30 or #31 or #32 or #33 or #34 or #35 or #36 or #37 | 624,298 |
| 39 | #7 and #38 | 2,227 |
| 40 | (pubstatusaheadofprint OR publisher[sb] OR pubmednotmedline[sb]) | 6,062,849 |
| **41** | **#39 and #40** | **475** |

[1] Centre for Reviews and Dissemination. *Systematic Reviews: CRD’s guidance for undertaking reviews in health care [Internet]*. York: University of York, 2009 [accessed 23.3.11] Available from: <http://www.york.ac.uk/inst/crd/SysRev/!SSL!/WebHelp/SysRev3.htm>

[2] Higgins JPT, Green S, eds. *Cochrane handbook for systematic reviews of interventions [Internet]*. Version 5.1.0 [updated March 2011]: The Cochrane Collaboration, 2011 [accessed 23.3.11]. Available from: <http://handbook.cochrane.org/>
